# Ultrasound-based evaluation revealed reliable postoperative knee stability after combined acute ACL and MCL injuries

**DOI:** 10.1186/s40634-021-00401-7

**Published:** 2021-09-15

**Authors:** Patricia M. Lutz, Louisa S. Höher, Matthias J. Feucht, Jan Neumann, Daniela Junker, Klaus Wörtler, Andreas B. Imhoff, Andrea Achtnich

**Affiliations:** 1 Department for Orthopedic Sports Medicine, Technical University Munich, Ismaningerstrasse 22, 81675 Munich, Germany; 2 Department of Diagnostic and Interventional Radiology, Technical University of Munich, Ismaningerstrasse 22, 81675 Munich, Germany; 3 Orthopädische Klinik Paulinenhilfe, Diakonie-Klinikum Stuttgart, Rosenbergstraße 38, 70176 Stuttgart, Germany

**Keywords:** MCL, ACL, Multiligamentous injuries, Ultrasound, Medial joint space, Valgus instability

## Abstract

**Purpose:**

Anterior cruciate ligament (ACL) injuries are often combined with lesions of the medial collateral ligament (MCL). The aim of this study was to evaluate treatment outcome of combined acute ACL and MCL lesions using functional US and clinical examination.

**Methods:**

Patients aged > 18 years undergoing primary ACL reconstruction with concomitant operative (group 1) or non-operative treatment of the MCL (group 2) between 2014 and 2019 were included after a minimum follow-up of 12 months. Grade II MCL injuries with dislocated tibial or femoral avulsions and grade III MCL ruptures underwent ligament repair whereas grade II injuries without dislocated avulsions were treated non-operatively. Radiological outcome was assessed with functional US examinations. Medial knee joint width was determined in a supine position at 0° and 30° of knee flexion in unloaded and standardized loaded (= 15 Dekanewton) conditions using a fixation device. Clinical examination was performed and patient-reported outcomes were assessed by the use of the subjective knee form (IKDC), Lysholm score, and the Tegner activity scale.

**Results:**

A total of 40 patients (20 per group) met inclusion criteria. Mean age of group 1 was 40 ± 12 years (60% female) with a mean follow-up of 33 ± 17 months. Group 2 showed a mean age of 33 ± 8 years (20% female) with a mean follow-up of 34 ± 15 months. Side-to-side differences in US examinations were 0.4 ± 1.5 mm (mm) in 0° and 0.4 ± 1.5 mm in 30° knee flexion in group 1, and 0.9 ± 1.1 mm in 0° and 0.5 ± 1.4 mm in 30° knee flexion in group 2, with no statistically significant differences between both groups. MCL repair resulted in lower Lysholm scores (75 ± 19 versus 86 ± 15; *p* < 0.05). No significant differences could be found for subjective IKDC or Tegner activity scores among the two groups.

**Conclusion:**

A differentiated treatment concept in combined ACL and MCL injuries based on injury patterns leads to reliable postoperative ligamentous knee stability in US-based and clinical examinations. However, grade II and III MCL lesions with subsequent operative MCL repair (group 1) result in slightly poorer subjective outcome scores.

**Level of evidence:**

Retrospective cohort study; Level III

## Introduction

Anterior cruciate ligament (ACL) injuries are in up to 35% of cases combined with lesions to the medial side of the knee [9, 31] and available evidence of treatment concepts for combined lesions is limited. Whereas anatomic reconstruction of the ACL (ACL-R) using an autologous tendon graft represents the current gold standard, treatment strategies for MCL injuries remain inconsistent [5, 8, 13, 19, 25, 36, 37]. Time of surgery, surgical techniques (repair or reconstruction), and indication as well as strategy of non-operative treatment are controversially discussed [4, 5, 8, 10, 28, 34, 40]. From a biomechanical point of view, there is growing evidence that MCL deficiency is a risk factor for ACL graft failure [1, 21, 23]. Therefore, postoperative MCL stability remains an important goal in the combined treatment of ACL and MCL injuries. Quantification of medial instability is commonly reported by clinical outcome, but radiological assessment is often missing. Functional ultrasound (US) examination can be used as a diagnostic tool to enhance postoperative radiological outcome measurement. Recently, mean values of medial joint space width in unloaded and standardized loaded conditions using a fixation device in healthy knees have been published [20]. However, results of functional US examinations for radiographic assessment following ACL-R and concomitant operative or non-operative treatment of the MCL are missing.

Therefore, the purpose of the present study was to evaluate the radiological and clinical outcomes after ACL-R with concomitant MCL repair or non-operative MCL treatment. It was hypothesized that surgical repair of the MCL would contribute to higher rates of valgus instability and worse functional outcomes when compared to non-operative treatment, since the indication for surgical treatment in the authors’ department was a high-grade MCL injury.

## Methods

This retrospective cohort study was conducted to evaluate the clinical and radiological outcome after anatomic ACL-R with or without early MCL repair in patients with combined ACL and MCL lesions. The study was approved by the institutional review board of the Technical University of Munich (235/19 S).

### Patient cohort

All patients presenting with combined acute ACL and MCL injuries at our institution between February 2014 and February 2019 were included in this study. Inclusion criteria were: subjects aged > 18 years, early ACL-R with autologous hamstring tendon and MCL repair (group 1) in the first two weeks after trauma or non-operative treatment of the MCL (group 2) for six weeks followed by staged ACL-R with autologous hamstring tendon. Diagnosis of combined ACL and MCL injuries were made using magnet resonance imaging and clinical examinations. Indication for MCL treatment was dependent on MCL grading according to Fetto and Marshall [9]. In grade II MCL injuries, an increased laxity at 30 degrees of flexion could be found, whereas in grade III MCL injuries an increased laxity at both, 0 and 30 degrees of flexion was present [9]. Grade II MCL injuries with dislocated tibial or femoral avulsions and grade III MCL injuries underwent ligament repair (group 1) due to the limited capacity to heal non-operatively [2]. Grade II MCL injuries without avulsions (partial ruptures) were treated non-operatively with a brace for six weeks with limited range of motion (ROM) and partial weight bearing on crutches (group 2).

Exclusion criteria for the present study were: further ligamentous or osseous injuries of the affected knee as well as previous injuries and surgical interventions on the other knee, and lack of German language skills.

Clinical notes of all patients were reviewed to collect demographic data.

### Operative technique

Prior to surgery all patients had undergone a thorough clinical and radiological (X-rays and MRI) examination to ensure ligament injuries to the ACL and MCL with or without concomitant meniscus lesions.

In case of concomitant meniscus lesions, meniscus suture systems (Arthrex, Naples, USA or Smith&Nephew, London, UK) were used or partial resection of meniscus was performed.

In both groups, an arthroscopic, anatomic single-bundle ACL technique with autologous hamstring graft was performed. The femoral tunnel was drilled via an anteromedial portal according to the diameter of the graft. A cortical suspension device (ACL tight-rope, Arthrex, Naples, USA) was used for femoral graft fixation. A K-wire was then placed in the center of the tibial ACL footprint and was overdrilled according to the diameter of the graft, creating the tibial tunnel. A bio-absorbable interference screw (Arthrex, Naples, USA) was used for tibial fixation.

In group 1, the medial collateral ligamentous structures were repaired by the use of suture anchors (Corkscrew 5,5 mm Biocomposite, Arthrex, Naples, USA) in case of femoral- or tibial-sided injuries with or without suture tape augmentation. Suture tape augmentation (FiberTape, Arthrex, Naples, USA) was used in grade III MCL injuries if medial collateral ligamentous structures were badly damaged resulting in poor tissue quality accompanied by limited success of isolated MCL repair by suture anchors. Additionally, MCL augmentation was performed in case of insufficient ligamentous stability after MCL repair intra-operatively.

### Postoperative rehabilitation

The postoperative protocol of group 1 consisted of 6 weeks of partial weight-bearing on crutches with limitation in ROM: in the first two weeks an active extension(ex)/flexion(flex) of 0°/20°/60°, in the next two weeks of 0°/10°/90°, and in the last two weeks of 0°/0°/90° was allowed. After six weeks, ROM was no longer limited. A brace (Medi M4, Medi Bayreuth, Germany) was provided for at least 12 weeks.

In group 2, partial weight bearing on crutches with the same limitations in ROM was allowed in the six weeks of non-operative treatment in a brace. After six weeks, ACL-R was performed. Postoperatively, partial weight bearing on crutches was allowed for two weeks and ROM was only limited if meniscus suturing was performed (ex/flex 0°/0°/90°).

Return to running on the treadmill and front crawl swimming was allowed after 6 weeks, trail running after 3 months, return to sport-specific training in both groups was allowed after 6 months and full return to contact and/or pivoting sports activities after at least 9 months postoperatively.

### Radiological evaluation

Functional US examinations were performed by two board-certified radiologists with at least 5 years of experience in musculoskeletal imaging at follow-up. All acquired images of the knees were evaluated on picture archiving and communication system PACS workstations (Sectra Medical Systems, Sweden).

### Ultrasound examination

**Fig. 1:**
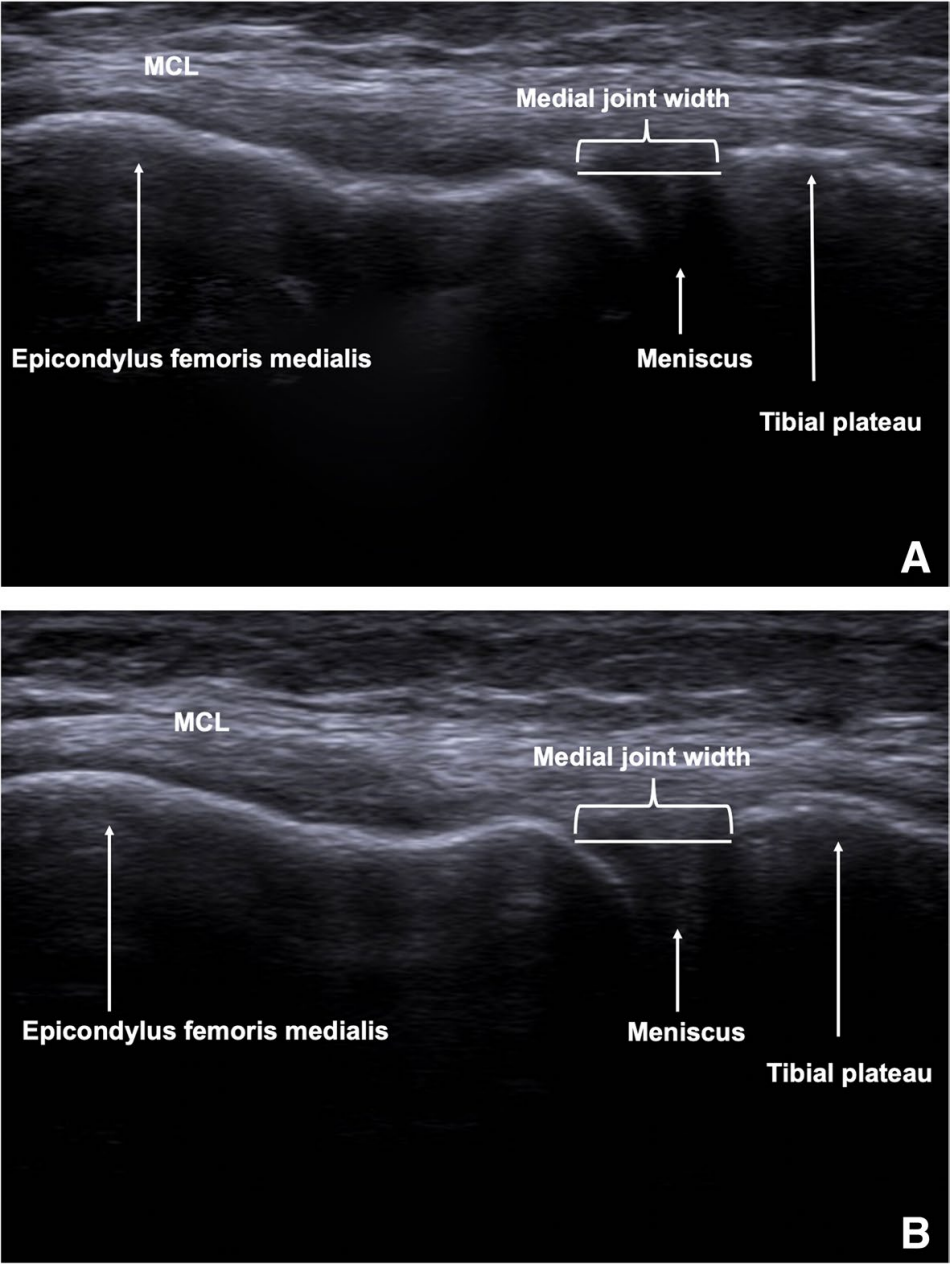
Ultrasound image of the medial joint space width in 30° knee flexion in unloaded condition (**1A**) and in standardized loaded condition (15 daN) with TELOS fixation device (**1B**). White arrows show medial femoral epicondyle, medial meniscus, and tibial plateau. A white line is positioned between femoral and tibial margins where medial joint width can be measured in millimeters. MCL, medial collateral ligament

For evaluation of medial ligament laxity, the width of the medial joint space was assessed by ultrasound (ACUSON NX3 Ultrasound System, Siemens Erlangen, Germany) using a linear transducer (4.0–12.0 MHz, Maximum Field of View: 153 mm, Maximum Display Depth: 160 mm), placed in a longitudinal direction over the medial aspect of the knee. Subjects were positioned supine with extended leg in 0° with and without reproducible applied valgus stress (loaded condition) through a fixation device (TELOS, Wölfersheim-Berstadt, Germany) with 15 dekanewton (daN), and in a second step with a 30° bended knee with and without valgus stress [11, 20]. For standardized measurements, the medial epicondyle was palpated and the transducer was placed in the longitudinal direction. Since the presentation of the hyperechoic bony outline of femur and tibia has been considered as an important quality assessment for standardized measurement of medial joint width [42], the medial femoral epicondyle and the proximal tibial plateau were used as bony landmarks, as described in the literature (Fig. 1A and B) [11, 20]. The distance between corresponding points on the femoral and tibial articular margins was measured in millimeters (mm). All measurements were performed by a specifically trained orthopedic sports medicine resident (rater 1). Intra- and interrater reliability was tested in 20 randomly assigned and blinded cases after an interval of six weeks by rater 1 and by one senior orthopedic surgeon (rater 2). Medial joint space width in 0° and 30° knee flexion was then compared between unloaded and standardized loaded conditions for each group. Furthermore, the mean change (delta **Δ**) of medial joint space width between unloaded and loaded conditions for each state of flexion and side-to-side differences of the average change (delta **Δ**) were compared between both groups.

### Clinical examination and patient-reported outcome measures

All subjects underwent standardized clinical examination of the knee. The International Knee Documentation Committee (IKDC) valgus subscore was used for objective MCL stability assessment. Meniscus was evaluated by joint space tenderness and Steinmann test [35]. Patient-reported outcomes were measured with Visual Analogue Scale for pain (VAS), the subjective knee form of the IKDC, the Lysholm score, and the Tegner activity scale at follow-up [14, 38].

### Statistical analysis

Statistical analysis was performed by use of SPSS software (IBM, Armonk, New York, USA) and Microsoft Excel Version 2019 (Microsoft, Redmond, Washington, USA). For all statistical tests, *p* values less than 0.05 were considered as significant.

Descriptive statistics are presented as mean ± standard deviation (SD) with a measurement accuracy of one decimal. Kolmogorov–Smirnov univariate normality test was used for continuous variables to confirm data normality. Mean change of medial joint space width between unloaded and loaded conditions and side-to-side differences were compared between the two groups using paired t-tests. Group comparison was performed with Mann–Whitney U test and unpaired t-test, as appropriate.

Intraclass correlation coefficients (ICCs) were calculated to determine the intra- and interobserver reproducibility. ICC values > 0.9 were considered excellent, values between 0.8 and 0.9 were considered good and values < 0.8 were considered poor. An a priori power analysis based on the results of Zaffagniniet al*.* was performed with G*Power (Erdfelder, Faul, Buchner, Lang, HHU Düsseldorf, Düsseldorf, Germany) [41]. The authors reported a mean medial joint space opening (side-to-side difference of medial joint opening with and without stress applied) measured with Telos valgus stress radiographs of 0.9 mm (SD 0.7) after isolated ACL-R, and of 1.7 mm (SD 0.9) after ACL-R combined with non-operative treatment of a grade II MCL lesion [41]. The power analysis revealed a sample size of 20 knees in each group at an assumed effect size of 0.81 in order to achieve a statistical power of 0.8.

## Results

A total of 40 patients were included. Twenty had an ACL-R with concomitant early MCL repair, and 20 had staged ACL-R with non-operative treatment of the MCL. Significant differences with regard to demographics were only observed for sex and MCL grading.

Patient demographics of the total study group are shown in Table 1.

**Table 1:** Patient demographics of the total study group. Continuous variables are shown as mean ± standard deviation (range), categorical variables are shown as percentages

	**Group 1**	**Group 2**	***p* value**
**Number of patients, n**	20	20	
**Follow-up (months)**	33.0 ± 16.7 (13–68)	34.3 ± 15.2 (12–52)	n.s
**Age (years)**	39.8 ± 12.1 (20–55)	33.1 ± 8.1 (23–60)	n.s
**Sex, n (%)**			
Male	8 (40%)	16 (80%)	< 0.05 ^a^
Female	12 (60%)	4 (20%)	< 0.05 a
**BMI (kg/m**^**2**^**)**	25.0 ± 5.7 (18.3–42.4)	27.4 ± 5.6 (20.1–39.4)	n.s
**Laterality, n (%)**			
Right	12 (60%)	6 (30%)	n.s
Left	8 (40%)	14 (70%)	n.s
**MCL grading, n (%)**			
Grade II	8 (40%)	20 (100%)	< 0.001 ^a^
Grade III	12 (60%)		
**MCL repair, n (%)**			
Anchor	14 (70%)		
Anchor + tape	6 (30%)		
**Meniscus-Suturing, n (%)**			
Lateral meniscus	9 (45%)	5 (25%)	n.s
Medial meniscus	0 (0%)	1 (5%)	n.s
Lateral and medial meniscus	4 (20%)	3 (15%)	n.s

*BMI*, body mass index; *MCL*, medial collateral ligament; *n.s.*, not significant

^a^Statistically significant difference between both groups

### Postoperative complications

In total, three patients suffered from postoperative stiffness with limitations to ROM because of arthrofibrosis. Re-arthroscopy with the aim of arthrolysis was necessary to regain full ROM after 5 and 7 months (group 1), and after 3 months (group 2).

### Clinical outcome

Group comparisons of the patient-reported outcome scores are shown in Table 2. With regard to the intervention, patients with MCL repair showed significantly higher VAS values at rest and lower Lysholm score results, whereas no significant difference was observed for IKDC subjective score and Tegner activity score. The mean return-to-sports time in group 1 was 22.3 ± 14.7 (range, 6–60) weeks, whereas in group 2 the mean return-to-sports time was 20.2 ± 8.8 (range, 6–36) weeks (n.s.).

**Table 2:** Results of outcome scores

	**Group 1**	**Group 2**	***p* value**
**VAS**^**a**^			
Rest	1	0	< 0.001^b^
Move	2	1	n.s
**IKDC**	78.0 ± 16.5 (49.4 – 99.0)	85.1 ± 12.8 (46.0 – 99.0)	n.s
**Lysholm score**	74.5 ± 18.5 (29.0 – 100.0)	85.6 ± 14.8 (37.0 – 100.0)	< 0.05^b^
**Tegner activity scale**^**a**^	4	5	n.s

Continuous variables are shown as mean ± standard deviation (range)

*VAS* Visual Analogue Scale (pain), *IKDC* International Knee Documentation Committee, *n.s.* not significant

^a^Values are median

^b^Statistically significant difference between both groups

### Clinical examinations

Concerning the ACL, one patient of group 2 showed positive pivot-shift test. Lachman test was positive in one patient of group 1 and in three patients of group 2. Valgus stress testing manually revealed 4 patients with IKDC grade B in 0° and in 7 patients with IKDC grade B in 30° knee flexion of group 1. All patients of group 2 showed IKDC grade A concerning valgus stability.

### Ultrasound examinations

Excellent intrarater reliability was observed for all measurements. The ICC values were 0.96 for unloaded states and 0.95 for loaded states. Interrater reliability was excellent with ICC values of 0.94 for unloaded and 0.95 for loaded states, respectively.

Results of US measurements are summarized in Table 3. First, the average change of medial joint space width between unloaded and standardized loaded conditions in 0° and 30° of knee flexion is shown. Second, side-to-side differences of the average change of medial joint space width between unloaded and standardized loaded conditions in each degree of flexion is explained. No statistically significant differences between both groups could be found.

**Table 3:** Analysis of medial joint space width in Ultrasound examinations for groups (mean ± SD); average change (Δ) between unloaded and loaded conditions in 0° and 30° knee flexion, as well as side-to-side difference of the average change (Δ) in 0° and 30° knee flexion

	*Δ of medial joint space width (mm) in 0°*	*Δ of medial joint space width (mm) in 30°*	*Side-to-side difference (mm) in 0°*	*Side-to-side difference (mm) in 30°*	*p value*
*Group 1*	2.3 ± 1.2	2.3 ± 1.3	0.4 ± 1.5	0.4 ± 1.5	*n.s*
*Group 2*	2.1 ± 0.7	2.1 ± 1.1	0.9 ± 1.1	0.5 ± 1.4	*n.s*

*mm* millimeter, *SD* standard deviation, Δ average change of medial joint space width between unloaded and loaded conditions, *n.s.* not significant

## Discussion

The most important finding of the present study was that comparable results regarding radiological quantification of MCL laxity can be achieved after operative or non-operative treatment of grade II and grade III MCL injuries when concomitant ACL injury is treated with reconstruction. After a mean follow-up of 34 months acceptable clinical and radiological outcomes could be found for both groups. Further findings were that patients after non-operative MCL treatment reached significant higher Lysholm scores, as well as superior IKDC and Tegner activity scores, although not reaching statistical significance.

Concerning the radiological method, functional US examination of medial structures of the knee has been shown to be a suitable method to describe medial joint space width [11, 17, 20, 30, 33]. In the present study, a fixation device was used to allow standardized and reproducible measurement of the medial joint space width [11, 20].

Mean side-to-side differences after combined ACL and MCL injuries were < 1 mm in both groups in the present study. In a systematic review, DeLonget al*.* reported an average side-to-side difference of 1.3 ± 0.9 mm after MCL repair [6]. Similar to our study, no significant side-to-side differences between groups after nonoperative grade II-III MCL treatment or MCL repair could be found by the use of valgus stress radiographs in previous research [12, 25, 41]. In our study, the average change (Δ) between unloaded and loaded conditions in 0° and 30° knee flexion was 2.3 mm in group 1 and 2.1 mm in group 2. Previous US examinations of healthy knee joints showed similar results [11, 20, 33]. The US results of the present study revealed that valgus stability after combined ACL and MCL injuries could be completely restored in both groups.

Since 100% of both groups were identified with IKDC grade A or B when valgus stability was tested manually in clinical examination, restoration of valgus stability could be confirmed. In general, findings of improved valgus stability were similar to those of previous research after different MCL treatment approaches [6, 7, 10, 12, 15, 16, 18, 19, 24, 27, 41].

Considering that combined ACL and MCL injuries mostly affect the active and young to middle-aged patient population, an important purpose of different treatment approaches is not only to achieve good to excellent objective ligamentous stability, but also subjective outcome scores and activity levels. In our total cohort, pain did not seem to play a major role postoperatively (VAS 0–2).

The presented findings revealed lower subjective outcome scores after MCL repair as compared to non-operative MCL treatment. This is in line with recent results of Westermannet al., who reported superior patient reported outcomes after non-operative grade III MCL injuries compared to operative MCL treatment in combined ACL and MCL injuries [39]. With regard to the subjective IKDC score, superior outcomes (94 and 89) were reported by *Canata *et al*.* and *Desai *et al*.* after ACL-R and MCL repair [3, 7]. Multiple authors reported good to excellent Lysholm scores after ACL-R and MCL repair [3, 6, 7, 18, 27], whereas only fair results could be found in the presented study. MCL repair was therefore not only seen to result in excellent objective outcomes, but was also associated with lower patient-reported outcome scores as compared to the non-operative MCL group.

Tegner activity scale outcomes of our entire cohort showed no statistically significant differences between the two groups and therefore corresponds to the observations of several authors who reported similar results [6, 19, 29].

Comparable to previous research [24, 29, 39], non-operative MCL treatment combined with ACL-R resulted in good Lysholm and subjective IKDC outcomes. As stated by Halinen et al. [12], a 10-point difference in the Lysholm score can be set clinically significant. This leads to the assumption that the impact of a more invasive surgical intervention (ACL-R and MCL repair) leads to clinically significant differences in subjective outcomes compared to ACL-R and non-operative MCL treatment.

Reasons for subjective lower scores after ACL-R and MCL repair are likely that higher grade MCL lesions resulted in marked extra-articular soft tissue injury. Symptoms could be associated with damaged MCL structures that are not addressed sufficiently by MCL repair. Hence, injuries to deep MCL structures may explain the lower subjective scores of the MCL repair group found in the present study.

MCL repair during ACL-R has been described as important risk factor for loss of knee function [26]. Postoperative stiffness due to arthrofibrosis in our cohort occurred in 2 patients of group 1 (10%) and in one patient of group 2 (5%) and was therefore lower as in previous research of Westermann et al*.* [39]. This may be attributed to ACL-R, as postoperative limited ROM is the most common complication [22, 32], which was supported by current findings. However, in 3 of 3 patients, re-arthroscopy with arthrolysis was clinically successful with normal ROM postoperatively.

Along with certain strengths, there are some limitations of this study. First, an experienced clinician examined all patients but was not blinded to this study. No specific measurements were taken to reduce bias. Second, the cohort included inhomogeneous injury patterns concerning the MCL. However, this fact represents this patient cohort. Third, additional meniscus injuries were not excluded from this study. Fourth, the study design was retrospective. Fifth, although all functional US examinations were performed in a standardized fashion by experienced radiologists, US remains an operator-dependent imaging method. Furthermore, the average final follow-up (33 months in group 1 and 34 months in group 2) might not be sufficient to evaluate the long-term success rate after combined ACL and MCL injuries. Further research is therefore indicated.

## Conclusion

A differentiated treatment concept in combined ACL and MCL injuries based on injury patterns leads to reliable postoperative ligamentous knee stability in US-based and clinical examinations. However, grade II and III MCL lesions with subsequent operative MCL repair (group 1) result in slightly poorer subjective outcome scores.

## Data Availability

All raw data (anonymized) is available from the corresponding author on request.
